# Warming mitigates root exudate-induced priming effects via changes to microbial biomass, community structure, and gene abundance

**DOI:** 10.1093/ismejo/wrag002

**Published:** 2026-01-15

**Authors:** Nikhil R Chari, Kristen M DeAngelis, Arturo A Aguilar, A Li Han Chan, Grace A Burgin, Serita D Frey, Benton N Taylor

**Affiliations:** Department of Organismic and Evolutionary Biology, Harvard University, Cambridge, MA 02138, United States; Department of Microbiology, University of Massachusetts, Amherst, MA 01003, United States; Department of Organismic and Evolutionary Biology, Harvard University, Cambridge, MA 02138, United States; Department of Microbiology, University of Massachusetts, Amherst, MA 01003, United States; Department of Plant Biology, University of Massachusetts, Amherst, MA 01003, United States; Department of Organismic and Evolutionary Biology, Harvard University, Cambridge, MA 02138, United States; Department of Natural Resources and the Environment, University of New Hampshire, Durham, NH 03824, United States; Department of Organismic and Evolutionary Biology, Harvard University, Cambridge, MA 02138, United States

**Keywords:** soil carbon, soil organic matter, stable isotope probing, rhizodeposition, temperature, decomposition

## Abstract

Root exudation, the export of soluble carbon compounds from living plant roots into soil, is an important pathway for soil carbon formation, but high rates of exudation can also induce rapid soil organic matter decomposition – a phenomenon known as the priming effect. Long-term soil warming associated with climate change could alter exudation rates and impact soil microbes by changing soil carbon chemistry. We hypothesized that warming-induced changes to exudation rate combined with direct effects of long-term warming on soil microbial communities would regulate the microbial priming effect. We tested this hypothesis with an artificial root exudate experiment using intact soil cores from a long-term soil warming experiment in a temperate forest. We found that chronic soil warming did not alter soil carbon formation from exudates, but did reduce the exudate-induced priming effect; exudation caused greater soil carbon loss in unwarmed than warmed soils. We used DNA stable isotope probing with 16S ribosomal RNA gene and shotgun metagenomic sequencing to determine whether long-term warming affected which microbes consume ^13^carbon-labeled artificial exudates. We found significant differences in bacterial community composition and relative gene abundances of ^13^carbon-enriched compared to natural abundance DNA. Both soil bacterial community composition and specific enzyme-coding gene families were strongly correlated with soil carbon priming in unwarmed treatments, but these effects were absent in warmed treatments. Our results suggest that the root exudate-induced priming effect is mediated by microbial biomass, community structure, and gene abundance, and that chronic warming reduces the priming effect by altering these microbial variables.

## Introduction

Warming global temperatures are shifting the balance of carbon (C) fluxes into and out of the soil [1, 2], and one C flux that is particularly susceptible to change with warming is root exudation—the export of low-molecular weight C compounds from living plant roots into soil—estimated at 10–20 Pg C y^−1^ globally [3]. Root exudates are non-structural C compounds that can exhibit rapid changes in production rates along diurnal [4] and seasonal timescales [5]. If warming enhances photosynthetic rates, increased plant production of non-structural C could drive higher rates of root exudation. Indeed a positive exudation response to warming has been observed in several climate warming experiments [6–8]. From a belowground perspective, root exudation is often viewed as a plant nutrient acquisition strategy for organic nitrogen (N) and phosphorus (P) [9, 10]. For this reason, if soil warming enhances microbial N and P mineralization rates and plant N and P availability, then root exudation rates could be reduced, and this effect has also been observed in some warming experiments [11, 12]. To understand how root exudates mediate warming effects on soil C, we need to understand both the responses of exudation rates and how exudates influence soil microbial processes in warmer soils.

The root exudate-induced priming effect refers to the process by which exudate C inputs stimulate enhanced microbial decomposition rates of native soil organic matter (SOM), resulting in soil C loss [13, 14]. Priming tends to increase as exudation rates increase, and high enough exudation rates can induce priming effects large enough to result in net soil C loss [14, 15]. Depending on whether root exudation rates increase or decrease in response to warming, the priming effect could be enhanced or reduced, respectively. However, priming effects rely not only on the amount of substrate input but also on the efficiency and diversity of soil microbial decomposers deriving energy from the exudates [13, 16]. Thus, warming could conceivably impact the root exudate priming effect in at least two ways: by altering the amount of root exudates or by altering the soil microbial community. Depending on if and how warming affects the soil microbial community, the root exudate priming effect could be compounded, reduced, or unchanged by warming.

In this study, we designed a ^13^C-labeled artificial root exudate experiment using intact soil cores from a long-term soil warming experiment to ask if and how the microbial community would modulate the root exudate priming effect. We hypothesized that warming would reduce the root exudate priming effect via several microbially-mediated mechanisms. First, soils exposed to long-term warming could have reduced total microbial biomass, which may be a result of depleted SOM stocks [17] or reduced quality of SOM available to soil microbial heterotrophs [18]. Chronic warming could reduce the priming effect by limiting the number of microbes available to decompose soil C. Second, warming has been shown to reduce the expression of carbohydrate active enzyme genes (CAZymes or CAZy genes) responsible for SOM decay [19]. Microbes producing fewer CAZymes would theoretically decompose less SOM and induce a smaller priming effect. Finally, warming could also affect soil microbial community composition [20, 21], although we did not have an *a priori* expectation for how this would impact the priming effect.

We also used DNA stable isotope probing to test the hypothesis that the priming effect would be carried out largely by bacteria specifically consuming exudate C. This result would be consistent with the theory that exudate C serves as an energetic source for soil bacteria to enhance SOM decomposition rates [22, 23]. Alternatively, priming could be dominantly carried out by bacteria which do not consume exudate C and must thus mineralize more native soil organic carbon (SOC) to remain competitive; this would be consistent with theory that the priming effect is derived from competition between bacteria specializing on different C sources [24]. Or, exudate uptake could induce negligible effects on the bacterial community, which would indicate that most bacteria consume exudate C and the priming effect is not dependent on a subset of the bacterial community.

**Figure 1:**
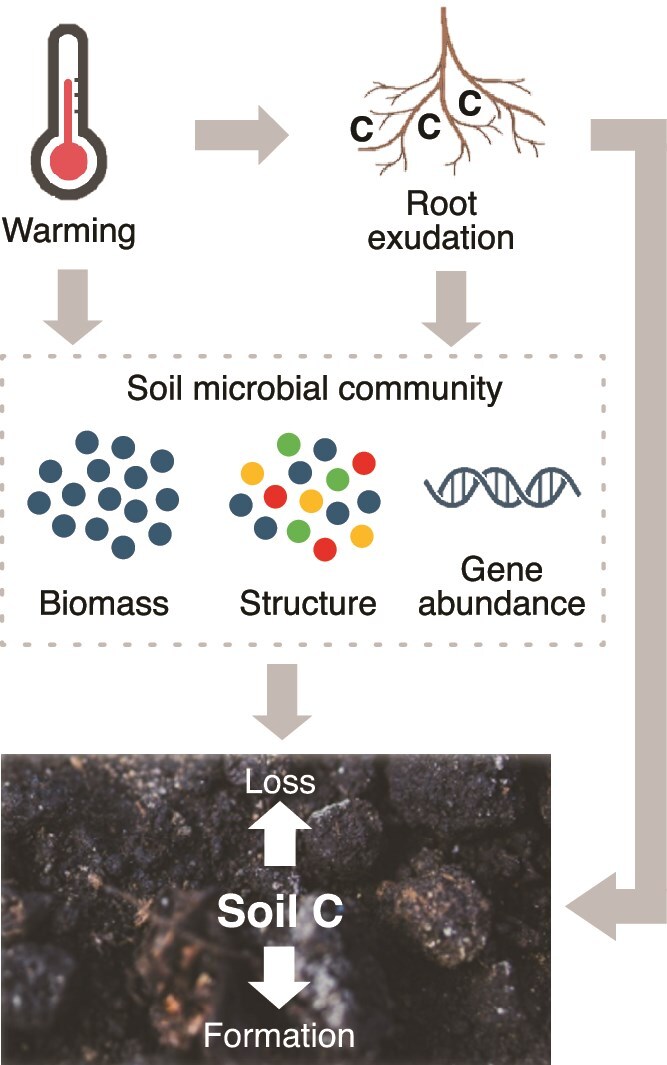
Schematic illustrating the relationships tested in this experiment. Here, root exudation can impact soil C formation and loss either directly or indirectly via the soil microbial community. Warming can impact root exudate effects on soil C dynamics by impacting the root exudation rate or the soil microbial community. Parts of this figure were created in BioRender.

To quantify the microbial priming effect, we measured pore-water C (i.e. the amount of SOC solubilized by exudate addition) as an indicator of the amount of priming induced by root exudates, as well as soil CO_2_ respiration and the change in soil C over time. We also measured soil microbial biomass, and we used 16S ribosomal RNA gene amplicon and shotgun metagenomic sequencing to determine bacterial community composition and functional gene relative abundance, respectively. We hypothesized that microbes consuming exudates would drive the root exudate priming effect, and that warming would reduce this effect by decreasing microbial biomass, altering bacterial community structure and altering microbial gene abundances, including by reducing CAZy gene abundance (Fig. 1).

## Materials and methods

### Experimental design

On July 26, 2021, we collected intact soil cores (2.5 cm diameter, ~8 cm depth mineral soil) from the Barre Woods long-term soil warming experiment at Harvard Forest (42° 28′ N, 72° 10′ W). Barre Woods is a temperate hardwood forest stand dominated by *Quercus rubra*, *Quercus velutina*, and *Acer rubrum* with sandy loam soils best characterized as Typic Dystrudepts [25]. Mean weekly air temperature at the site varies from a high of 20°C in July to a low of −6°C in January [26]. The soil warming experiment was established in 2001 as two large 30 × 30 m forest plots, one of which was heated to 5°C above ambient by buried warming cables [25]. Thus, at the time of sampling, the soils in the warmed plot had been exposed to 20 y of +5°C warming. Soil temperature in the unwarmed plot ranges from ~10 to 20°C during the growing season, with the +5°C warming plot thus ranging from 15 to 25°C [26].

We randomly collected 45 experimental cores within both the warmed and unwarmed plots (90 total samples). We removed the organic horizon and immediately placed the mineral soil of each of these cores in a 50 ml centrifuge tube with four holes drilled in the sides and one in the lid for aeration and moisture loss. For each experimental core, we also collected a replicate core directly next to the experimental core for baseline measurements of moisture and SOC.

We refrigerated the cores at 4°C overnight and set up the artificial root exudate experiment on July 27, 2021. We inserted a 5 cm microporous Rhizon sampler (Rhizosphere Research Products, Wageningen, NL) “artificial root” fully into each core through the hole in the lid of the centrifuge tube. Each artificial root was connected to a separate syringe for delivery of artificial root exudates. Exudate solutions were delivered using a manual pump system at a rate of 1 ml d^−1^ from July 27–August 23, 2021. The experiment took place at room temperature (~20°C), so the unwarmed and warmed soil cores were exposed to the same temperature over the course of the experiment, and only the effects of long-term warming are assessed. Such a temperature is commonly experienced on a daily basis in the summer by soils in both the unwarmed and warmed plots in this experiment. A similar experimental design is previously described [15].

Artificial exudate solutions were prepared using a 4:1 mass ratio of succinic acid to glucose, as organic acids and simples sugars are generally among the most commonly occurring root exudate compounds [27–30], and were ^13^C-labeled to δ^13^C = +3000‰. Three exudation treatments were applied factorially across both warming treatments. A low exudate rate treatment delivered exudate C at a rate of 7 μmol C cm^−2^ artificial root surface area d^−1^ [15], and a high rate treatment delivered exudate C at a rate of 11.27 μmol C cm^−2^ d^−1^. The third treatment did not include any exudate C, but water was still delivered at a rate of 1 ml d^−1^, as in the other treatments. All three treatments also included low concentrations of dissolved salts for osmoregulation per Keiluweit et al. 2015 [31].

### Biogeochemical measurements

Over the course of the incubation, we weighed the experimental cores weekly to account for soil moisture change. Moisture was not adjusted from field conditions other than by artificial exudate additions. Moisture increased over the course of the experiment due to the exudate additions by an average of 0.3 g water g^−1^ dry soil, but there were no treatment effects on the change in moisture. On the last day of the experiment, we measured CO_2_ flux from the soil cores. We placed each centrifuge tube (with the cap off and “artificial root” removed) in a sealed jar fit with a rubber septum for 3 h, after which we sampled 30 ml of headspace gas through the septum with a syringe and stored it in a pre-evacuated 20 ml headspace vial for measurement of CO_2_ and ^13^CO_2_. Total CO_2_ and ^13^C-CO_2_ respiration and pore-water C and ^13^C were measured using a GasBench isotope ratio mass spectrometer (IRMS; Thermo Fisher Scientific, Waltham, MA, USA) at the Yale Analytical and Stable Isotope Center, New Haven, CT, USA. Immediately following the end of the experiment, the Rhizon samplers were removed, acid washed, randomized, and reused as pore-water samplers for pore-water C, ^13^C, and aluminum (Al). Pore-water Al was measured via a spectrophotometric assay [15, 32] at the Arnold Arboretum of Harvard University, Boston, MA, USA. Because the Rhizon samplers also served as artificial roots we randomized the samplers before collecting pore-water to redistribute any ^13^C from the experiment. Thus, our measurement of pore-water ^13^C captures treatment effects, rather than exact values.

After the experiment, we homogenized the experimental soil cores and reserved 20 g wet soil to measure microbial biomass C via chloroform fumigation and total organic C in fumigated and unfumigated extracts was measured using a TOC analyzer (Shimadzu, Kyoto, JP) [33]. We also reserved 0.25 g soil for DNA extraction. The remaining soil was air-dried and then particle-size fractionated, as were the replicate cores. We performed particle size fractionation to separate soil into mineral-associated (<54 um) and particulate (54–2000 um) size fractions [34]. Briefly, air-dried soils were sieved (<2 mm) and shaken at 180 RPM with a dispersant (sodium hexametaphosphate) and glass beads for 18 h before being passed sequentially through 2 mm and 54 μm sieves. Mineral-associated organic matter (MAOM) C and ^13^C were measured using an elemental analyzer-IRMS (Thermo Fisher) at the Cornell Stable Isotope Lab, Ithaca, NY, USA. We measured MAOM C formation from exudates using a two-pool isotope mixing model as previously described [15]. We measured the net change in MAOM C by subtracting initial MAOM C (%) in the replicate cores from final MAOM C in the paired experimental cores.

### DNA extraction and stable isotope probing

We extracted DNA from 0.25 g homogenized wet soil per sample using a DNeasy PowerSoil Pro kit (Qiagen, Hilden, GE). For the samples receiving ^13^C-labeled exudates, we used density gradient fractionation to separate ^13^C-enriched DNA in soil microbes consuming exudate C from natural abundance DNA [35]. Details of this method may be found in the supplement. Briefly, we pooled “heavy” (^13^C-enriched) and “light” (natural abundance) fraction samples based on a density cutoff. Thus, our pooled heavy and light samples do not represent “pure” ^13^C-DNA and natural abundance DNA, respectively, but differences between the heavy and light fraction are representative of differences between microbes consuming exudate C, and those not.

### DNA sequencing and analysis

We performed 16S rRNA gene amplicon sequencing to characterize bacterial community composition and shotgun metagenomic sequencing to characterize gene abundance. To characterize community composition, we constructed libraries using V4 amplification primers for barcoded 16S ribosomal RNA genes 515F (5′- GTGYCAGCMGCCGCGGTAA-3′) and 805R (5′- GGACTACNVGGGTWTCTAAT -3′) [36]. A total of 58 samples (23 light, 23 heavy, 12 not receiving exudates) were paired end sequenced (2 × 158bp) on a MiSeq System (Illumina). Community analysis focused on bacteria because bacteria are important short-term assimilators of exudate C [37], and our DNA-SIP and amplicon sequencing workflows were optimized for bacterial communities.

To characterize gene abundance, we constructed shotgun metagenomic sequencing libraries using the NEBNext Ultra II kit (New England Biolabs, Ipswich, MA, USA) on a subset of samples (n = 31, 11 light, 12 heavy, eight not receiving exudates). These libraries were paired end sequenced (2 × 151bp) on a NextSeq System (Illumina). All sequencing was performed by the Genomics Resource Laboratory at the University of Massachusetts, Amherst, USA. The average sequencing depth was 3.8 Gb/sample. Using shotgun sequence data, we assembled a table of enzyme commission (EC) gene families. We normalized EC abundances to reads per kilobase per million reads to account for sequencing depth and gene length. Information on processing of sequence reads may be found in the supplement. Because our sequencing approach for community composition focused on bacteria (16S rRNA gene amplicon sequencing) whereas shotgun sequencing for gene abundances would include fungal genes, we use the term “bacterial” when referring to community structure and “microbial” when referring to gene abundances.

### Statistics

For biogeochemical measurements, we used ANOVA models with the response variable (e.g. soil total C and ^13^C, pore-water C and ^13^C, microbial biomass C, or CO_2_ respiration) as a function of warming and exudation rate treatments, and their interaction. In the case of significant treatment effects, we performed Tukey’s Honest Significant Difference tests to assess differences between groups. In the case of significant interactive effects, we considered only the interaction. In the absence of interactive effects, we considered the main effects separately.

For microbial amplicon sequence data, we used NMDS ordination within *phyloseq* to compress bacterial community composition to two NMDS axes [38]. We performed PERMANOVA analysis with the libraries *vegan* and *pairwiseAdonis* to determine statistical differences in bacterial community composition between treatment groups [39, 40]. We determined which bacterial taxa responded most strongly to treatments using the *simper* function from *vegan* [40].

For the EC data derived from shotgun sequencing, we used ANOVA models to assess the response of EC and CAZy gene families to treatments. We performed NMDS and PERMANOVA to assess variation in CAZy EC composition and overall EC composition between treatments. We rotated NMDS ordinations so pore-water C loaded onto the primary NMDS axis to determine if these microbial variables were related to the root exudate priming effect [41]. We performed weighted gene correlation network analysis (WGCNA) to cluster related ECs into module “eigengenes” and determine which ECs were most strongly related to pore-water C [42]. We used the Kyoto Encyclopedia of Genes and Genomes (KEGG) mapper tool to map these ECs to associated cellular pathways [43]. All statistical analyses were carried out in R [44].

## Results

### Soil C formation from root exudates

**Figure 2:**
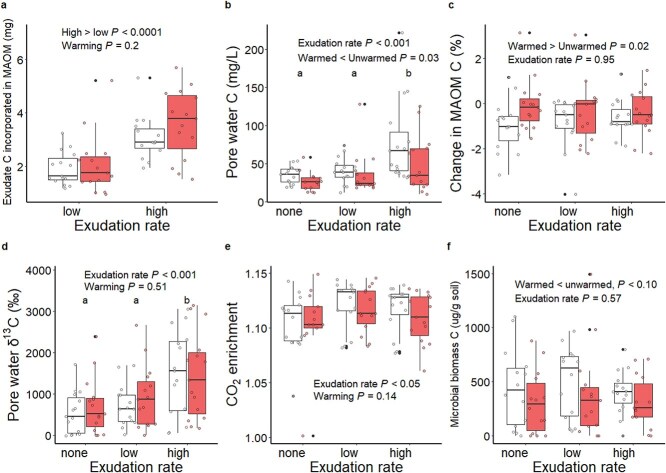
Effects of root exudation rate and warming on mineral-associated organic matter (MAOM) C formation (A), pore-water C (B), net change in MAOM C (measured as difference in MAOM C between experimental and replicate cores) (C), pore-water ^13^C (D), CO_2_ respiration relative to atmospheric concentration (E), and microbial biomass c (F). Boxes from “unwarmed” treatments are white and boxes from “warmed” treatments are red. different letters represent groups that are significantly different from each other by Tukey’s HSD (*P* < .05).

By tracing the ^13^C exudate label into the mineral-associated size fraction, we observed that higher rates of exudation led to more MAOM C formation (*P* < .001; Fig. 2A). Warming had no effect on MAOM C formation. We observed a similar pattern with pore-water δ^13^C, with greater δ^13^C values as exudation rates increased (*P* < .001; Fig. 2D) but no effect of warming. We expect the amount of residual exudate C to be very low at the concentrations used in our experiment [45], thus pore-water ^13^C likely reflects exudate C that has been consumed by microbes and turned over. These results show that warming did not affect soil C formation from root exudates.

### Soil C loss from root exudates

We measured pore-water C as an indicator of soil C loss due to the root exudate priming effect. Pore-water C increased with exudation rate (*P* < .001) but the magnitude of this effect was reduced by warming (*P* < .05; Fig. 2B, Fig. S7). In other words, warmed samples had consistently less soil C loss to the pore-water than unwarmed samples. In contrast, warming did not affect pore-water ^13^C, nor were pore-water C and ^13^C significantly correlated in any of the exudate treatments (Fig. S2), indicating that the reduction in pore-water C due to warming must be due to reduced native soil C rather than an effect of ^13^C-labelled exudate C. On average 7% and 28% of total pore-water C was derived from exudate C in the low and high rate treatments, respectively. We found no effect of exudation on the net change in MAOM C (ΔMAOM) over the course of the experiment (Fig. 2C), indicating that soil C formation and soil C loss due to root exudation were largely in balance. However, warmed soils had a less negative ΔMAOM than unwarmed soils (*P* < .05), meaning more MAOM C was retained over the course of the experiment in warmed compared to unwarmed soils. Measurements from replicate cores showed that MAOM C was similar between warming treatments at the start of the experiment, but MAOM C:N was higher in soils from the warmed plot (Fig. S8).

Several other variables matched the patterns observed in pore-water C and ΔMAOM, suggesting that exudation induced a priming effect that was reduced by warming. We found a significant interactive effect of warming and exudation rate on concentrations of pore-water aluminum (Al), such that pore-water Al was lower in warmed than unwarmed samples in soils receiving root exudates (Fig. S4), suggesting reduced dissolution rates of mineral-organic complexes in response to exudation in warmed soils. We also found that CO_2_ flux was higher in soils receiving exudates than those not (*P* < .05; Fig. 2E), and warming also appeared to reduce CO_2_ respiration, though not significantly (*P* = .14). Warming did not affect δ^13^CO_2_ (*P* = .82; Fig. S3), so any reduction in respiration due to warming was likely due to a reduction in the priming of native soil C rather than respiration of exudate ^13^C. Finally, we found that microbial biomass C was reduced in warmed soils, though this effect was marginal (*P* < .1; Fig. 2F).

### Soil bacterial community

**Figure 3:**
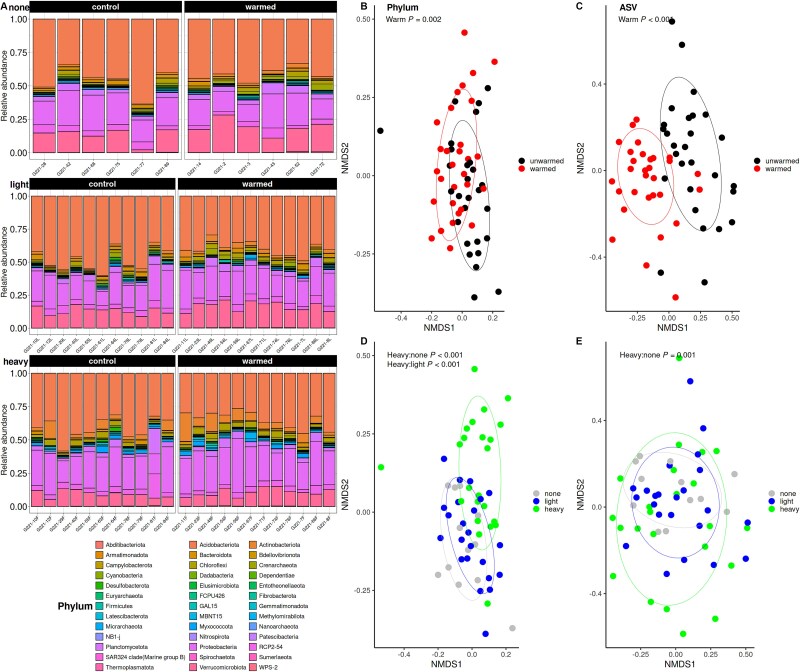
Differences in bacterial community composition between warmed and unwarmed treatments, and between microbes consuming enriched in exudate ^13^C DNA and microbes not enriched in exudate ^13^C. “None” refers to the treatment where no exudates were applied. differences in phylum relative abundance are shown in (A). NMDS ordinations were performed on phylum-aggregated (B, D) and ASV-aggregated (C, E) datasets. Points are colored by warming treatment in (B, C) and by exudate uptake in (D, E). “Heavy” refers to ^13^C enriched samples, “light” refers to natural abundance samples, and “none” refers to samples not receiving an exudate treatment.

We found that warming altered bacterial community composition at both the phylum and ASV levels (*P* < .01; Fig. 3A-C). Additionally, we used DNA stable isotope probing to separate bacterial communities into heavy fractions enriched in exudate ^13^C and light fractions not enriched in ^13^C. We found that bacterial community composition also varied depending on whether microbes were consuming exudate C (*P* < .01; Fig. 3A, D, E). Specifically, the heavy fraction microbes had significantly different phylum-level composition than both the light fraction microbes and microbes from control samples with no exudate input (*P* < .01). Heavy fraction samples also had different ASV composition than the control samples (*P* < .01), while the light fraction samples and control samples had statistically similar ASV composition (Fig. 3D, E). We also found a significant interaction effect between warming and exudation rate on bacterial community composition at the ASV level (*P* < .01), which was driven by a difference between low and high rate treatments in the warmed, but not unwarmed, samples (Fig. S5).

At the phylum level, warming drove a 7% decrease in relative abundance of *Acidobacteriota*, the largest phylum by relative abundance (*P* < .001; Fig. 3A). This was accompanied by increases in *Verrucomicrobiota* (3%; *P* < .05) and *Actinobacteriota* (2%; *P* < .05), and smaller increases in *Chloroflexi*, *Chrenarchaeota*, and several other phyla (*P* < .05; Table S1). The observed bacterial community responses could be due to adaptation to long-term soil warming or short-term acclimation to room temperature during the course of the artificial root exudate experiment. Because the room temperature during incubation (20°C) is commonly experienced *in situ* in both the warmed and unwarmed plots during the summer growing season, we believe these responses reflect long-term adaptation to warming rather than short-term acclimation.

Phyla consuming exudate C also had different relative abundances than those not, with *Proteobacteria* (the second most abundant phylum) enriched by 5% in the heavy relative to light fraction (*P* < .05; Fig. 3A). *Actinobacteriota* also increased in the heavy fraction by 4% (*P* < .001), while *Acidobacteriota* was reduced in the heavy fraction by 6%, though the decrease was marginally significant (*P* < .1). *Verrucomicrobiota* was reduced in the heavy fraction by 5% relative to the light fraction and 6% relative to the control samples, though only the difference from the controls was significant (*P* < .001). Several other phyla varied significantly at smaller abundances, between the heavy and light fraction (*P* < .05; Table S1). Only one phylum at moderate abundance varied significantly between control and light fraction samples (*Crenarchaeota*, *P* = .05), providing further support that the light fraction bacterial community not consuming exudate C was similar to the bulk soil community.

### Gene abundance

**Figure 4:**
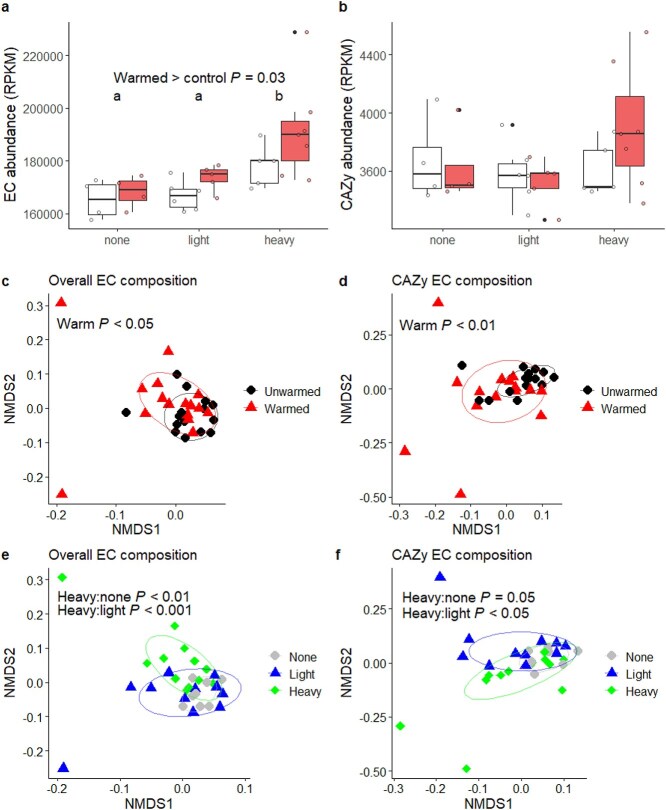
Response of EC gene families to warming and in response to exudate ^13^C uptake. Total abundance of ECs (A) and CAZy ECs (B) in reads per kilobase per million reads. Boxes from “unwarmed” treatments are white and boxes from “warmed” treatments are red. different letters represent groups that are significantly different from each other by Tukey’s HSD (*P* < .05). EC compositional response to warming (C) and exudate ^13^C uptake (D). CAZy EC compositional response to warming (E) and exudate ^13^C uptake (F).

From shotgun metagenomic sequence data, we found that the relative abundance of EC gene families increased in response to warming and was greater in the heavy fraction microbial community relative to both the light fraction and control treatments, indicating increased potential for enzymatic activity in bacteria consuming exudate C and under warming (*P* < .05; Fig. 4A). However, we did not find any effects of warming or rate treatments on the relative abundance of CAZy genes, and CAZy gene abundance did not vary with exudate uptake (Fig. 4B).

We observed effects of warming on the composition of both CAZy ECs and all ECs. We also found that the composition of both CAZy ECs and all ECs was different in microbes consuming exudate C. Specifically, the composition of ECs in general and CAZy ECs was significantly different between heavy fraction samples and both light fraction and control samples (Fig. 4C–F). Both general EC composition and CAZy EC composition were statistically similar between light and control fraction samples.

### Evidence that the bacterial community mediates the priming effect

**Figure 5:**
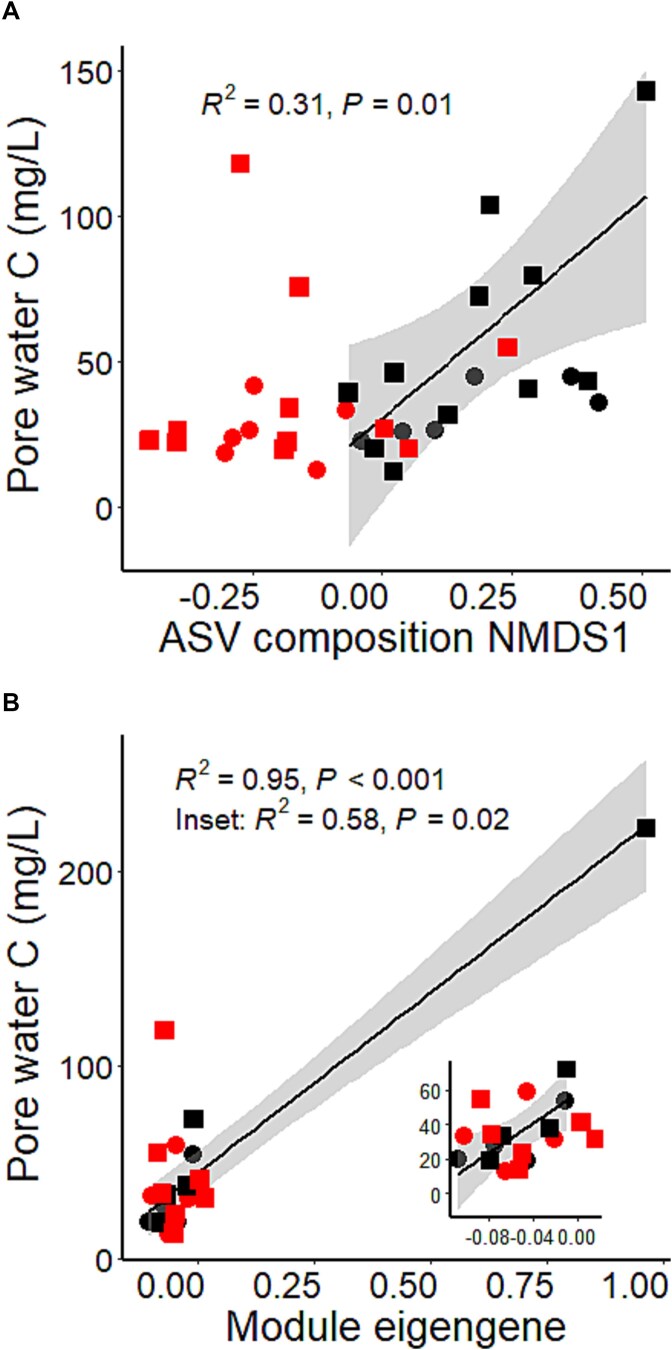
Relationships between ASV composition (A) and WGCNA module eigengene (B) with pore-water C. *R*^2^ and *P* values refer to correlations in the “unwarmed” (black) treatments. No correlations were found within the warmed (red) treatments. The inset panel in (B) includes only points with pore-water C < 100 mg L^−1^, to omit outsize influence of the point on the far right of the graph. Circles are samples from control treatments receiving no exudates, and squares are heavy fraction samples enriched in exudate ^13^C.

We found a strong relationship between bacterial community composition and pore-water C in the unwarmed, but not warmed, soils (Fig. 5A). The relationship between bacterial community composition and pore-water C was stronger with heavy fraction than light fraction samples (Fig. S6a).

We sought to determine which bacterial phyla drive the relationship between bacterial community composition and pore-water C in warmed versus unwarmed soils. The phyla *Myxococcota*, *Armatimonadata*, and *Thermoplasmatota* were strongly correlated with pore-water C in the unwarmed treatments (*P* < .001), though not in the warmed treatments. These phyla were not significantly reduced in abundance by warming, suggesting the warming effect on pore-water C may be due to a change in bacterial behavior rather than abundance, or by a change in bacterial community within phyla. Pore-water C was not significantly correlated with the primary NMDS axis of phyla composition, only ASV composition.

We used WGCNA to further determine which gene families are associated with the priming effect. We found that one module “eigengene” (representing an agglomeration of separate but correlated gene families) including 24 unique ECs was strongly correlated with pore-water C (*P* < .001). This module was not strongly correlated with pore-water ^13^C, nor were any other modules. This correlation was driven by samples in the unwarmed treatment; the module eigengene was not related to pore-water C in warmed treatment samples (Fig. 5B). Like the ASV effect, this effect was also driven by heavy fraction samples; when we replaced the heavy fraction with light fraction samples, the strength of the relationship was reduced (Fig. S6b). We also found that this module was positively correlated with the relative abundance of *Proteobacteria* in these samples (Spearman’s *ρ* = 0.47, *P* < .05). To confirm the warming effect, we performed WGCNA separately on samples from unwarmed and warmed treatments. We found one module with 67 unique ECs was strongly correlated with pore-water C in the unwarmed treatment, but no modules were correlated with pore-water C in the warmed treatment.

To further probe the function of genes related to priming soil C, we used the KEGG mapper tool to map the identity of the 67 ECs associated with pore-water C in the unwarmed samples to biological pathways [43]. We found these ECs were related to a diverse suite of biological pathways, including “Metabolic pathways” (37 ECs), “Biosynthesis of secondary metabolites” (16 ECs), “Microbial metabolism in diverse environments” (12 ECs), “Cysteine and methionine metabolism” (four ECs), and many others (Table S2). Only one of these 67 ECs was a CAZyme.

## Discussion

Here, we show that the priming effect is reduced under long-term soil warming and provide evidence that taxonomic and functional shifts of soil bacteria due to warming are related to this reduced priming effect. Using DNA SIP, we show that soil C loss due to priming is associated with the soil bacterial community consuming exudate C, but that long-term soil warming removes this association. Our results suggest that changes to bacterial community composition and function may drive reductions in the root exudate priming effect under warming.

### Warming mediates soil C loss, but not soil C formation, from root exudates

Root exudates can form soil C by being assimilated by microbes or binding to soil minerals. We found that higher rates of exudation resulted in more MAOM C formation, but warming did not affect MAOM C formation from root exudates (Fig. 2A). More MAOM C formation in response to more exudate C supply is intuitive and anticipated [15]. The absence of a warming effect on MAOM C formation suggests that microbial C assimilation and turnover rates remain similar in the warmed soils, and that warming does not impact the mineral surface area available for sorption of new C inputs [46].

In addition to forming soil C, exudates can also result in loss of pre-existing soil C via the destabilization of mineral-organic complexes and microbial priming effect [31]. Here, we confirm prior work showing that increased exudation rates increase soil C loss, suggest the priming effect is magnified at higher exudation rates [15]. Unlike soil C formation, which was not affected by warming, we found several lines of evidence showing that warming did impact soil C loss from root exudates, including reduced pore-water C (Fig. 2B), reduced pore-water metals (Fig. S4), reduced CO_2_ flux from soil (Fig. 2E), and decreased loss of MAOM C under warming (Fig. 2C). Reductions in the root exudate priming effect due to warming could be due to changes in the soil microbial community, changes in soil C chemistry under warming, or both.

Drought or soil drying under warming could reduce aqueous pore space needed for soil bacteria to live, potentially limiting the magnitude of the priming effect [47]. Moisture and temperature shifts brought on by soil warming have both been found to alter rhizosphere bacterial community composition [48, 49]. Furthermore, research at our field site suggests that long-term soil warming increases the chemical complexity and reduces decomposability of soil C [50, 51], which could reduce the potential for priming effects. Our initial measurements of higher MAOM C:N ratios in the warmed plot were consistent with the conclusion that soil C quality is reduced by long-term soil warming. Lower C quality could be a potential mechanism for the reduced priming effect we observed (Fig. S8). We sought to determine if warming would also impact microbial regulation of the priming effect.

### Microbial regulation of the priming effect and warming response

We found that soil microbial biomass was slightly reduced in the warmed soils (Fig. 2F), which could limit the potential for microbes to carry out the priming effect. This response of microbial biomass to warming aligns with several other measurements at this and adjacent field sites which found reduced microbial biomass under warming [17, 51, 52]. Nonetheless, although reduced soil microbial biomass may partially account for reduced soil C loss under warming, it cannot in and of itself explain the priming effect because total microbial biomass did not increase in response to exudation. Thus, it is more likely that exudates induce changes to the microbial community and/or the enzymes they produce which drive the priming effect. If these changes were less pronounced or absent under warming, that could explain the absence of a priming effect.

We used DNA stable isotope probing to separate ^13^C-enriched “heavy fraction” DNA (*i.e.* from microbes consuming exudates) from ^13^C-depleted “light fraction” DNA (*i.e.* from microbes not consuming exudates). We found that the bacterial community consuming exudates was taxonomically distinct from the general bacterial community (Fig. 3D,E). Broadly, we found that bacteria from the phyla *Proteobacteria* and *Actinobacteriota* were enriched in the heavy fraction, whereas *Acidobacteriota* and *Verrucomicrobiota* were reduced in abundance in the heavy fraction compared to the light fraction (Fig. 3A). *Proteobacteria* are often associated with rapid C mineralization whereas *Acidobacteriota* abundance is negatively associated with C mineralization rates [53], suggesting microbes consuming exudates are more likely to be C-mineralizing taxa likely responsible in part for the priming effect. But our results also suggest that bacterial community regulation of the priming effect and its response to warming occur at finer taxonomic levels. Specifically, we found that the bacterial ASV composition (but not the phyla composition) was strongly correlated with pore-water C (Fig. 5A), indicating that the bacterial community traits that may play a role in the priming effect are likely not deeply phylogenetically conserved, but may reflect traits that are more recently acquired or horizontally transferred.

We found the relationship between microbial community composition and soil C loss was driven by microbes consuming exudate C; the relationship was stronger with heavy fraction than light fraction samples (Figs 5, S6). However, we found this relationship was absent under warming, which leads us to conclude that warming altered the bacterial community to disfavor microbes capable of inducing a priming effect. The abundances of phyla *Myxococcota*, *Armatimonadata*, and *Thermoplasmatota* were strongly correlated with soil C loss in the unwarmed, but not warmed soils. *Armatimonadata* are oligotrophs with the ability to degrade complex C compounds [54], potentially allowing them to survive on exudate inputs applied in this experiment which did not include nutrients, and drive a soil C priming effect. *Thermoplasmatota* is an Archaeal phylum that includes methanogens [55], suggesting mineralization of soil C during methanogenesis as a potential priming effect mechanism. Our analysis of microbial community composition was restricted to bacteria and archaea and does not include fungal community composition. Several studies have found that warming does induce changes in fungal community composition, which likely feedback on soil C cycling [20, 56]. However, fungi may be more likely to rely on more complex, polymeric substrates than exudates as a C source [57], and our short-term study design and analytical approaches were best suited to detect bacterial community responses [36, 58]. In our taxonomic annotation of shotgun metagenomic data (which should also include fungi) we were only able to ID bacteria and archaea at the phylum level, suggesting that fungal ECs may play a relatively minor role in the priming effect.

We hypothesized that exudation could induce the priming effect by increasing the abundance of CAZy genes involved in SOM decomposition [59]. Although we did find that both warming and exudation increased the presence of EC gene families (indicating greater potential enzyme activity under warming and in microbes consuming exudates), neither of these treatments affected CAZy gene abundance specifically (Fig. 4A, B). Both warming and exudate uptake did significantly and independently impact the composition of both CAZy ECs and ECs overall (Fig. 4C–F), but only one of 67 ECs in the WGCNA module correlated with pore-water C was a CAZy EC. This was sterol 3beta-glucosyltransferase, which is a glycosyltransferase enzyme involved in transfer of glucose molecules. We suggest that the priming effect is not mediated by the abundance of CAZy ECs, but it may be mediated by later steps in CAZyme production, such as CAZy gene transcription or enzyme production itself [19]. Additionally, because root exudates are typically simple low-molecular weight compounds, they may not stimulate CAZyme production because they do not need CAZymes to be metabolized.

The 67 ECs potentially driving the priming effect corresponded to a diverse suite of metabolic pathways (Table S2), and these pathways may be avenues for further exploration of the microbial priming effect. Several ECs were involved in amino acid (cysteine and methionine) metabolism, suggesting that N mining could be a potential mechanism inducing the priming effect [60]. Other ECs were implicated in inositol phosphate metabolism; inositol phosphates are cell membrane molecules crucial in nutrient acquisition signaling [61]. The implicated functional genes suggest some importance of nutrient acquisition in driving the priming effect at a cellular level. Enhanced nutrient mineralization in the warmed soils may have helped decouple these relationships [62]. Furthermore, because initial MAOM C:N ratios were lower in the unwarmed plots (Fig. S8), mining for organic N may have been a viable nutrient acquisition strategy for microbes in the unwarmed plots, but not the warmed plots. The artificial exudates supplied in this experiment did not contain N or other nutrients; if nutrient acquisition is a mechanism causing the priming effect, changes to exudate stoichiometry could also result in varying microbial priming effect responses. Several genes were also associated with methane and sulfur metabolism, specifically methanogenesis, which could be due to the role of anaerobic Thermoplasmatota in driving the priming effect [63].

Our observation that the same ECs that corresponded to soil C loss in the unwarmed samples did not do so in the warmed samples suggests that changes to SOM chemistry over 20 years of warming may have reduced the capacity of microbes expressing these gene families to induce a priming effect [50]. Additionally, we found the positive relationship between the WGCNA module eigengene and pore-water C was driven by heavy fraction samples; as with the bacterial community, the correlation between the module eigengene and pore-water C weakened when these were replaced with light fraction samples. The module eigengene was also correlated with the relative abundance of *Proteobacteria* in the same samples, providing further evidence that fast-C-cycling *Proteobacteria*, which were also enriched in the heavy fraction samples, play a role in the priming effect. These lines of evidence suggest that microbes specifically consuming exudate C drove a priming effect and loss of soil C to pore-water, and that warming likely reduced this effect by altering several microbial variables including microbial biomass, bacterial community structure, and gene composition, as well as SOM chemistry.

Relationships between root exudation and the soil microbial community are bidirectional – signals from bacteria in the soil are also shown to influence exudation patterns [64]. However, by artificially manipulating presence/absence and rate of exudation in this experiment, we are able to explicitly determine the effects of exudation on soil microbial communities without the possibility of a bidirectional effect. Despite these advantages, artificial root exudate experiments are limited because they do not capture the complexity of root exudation in nature. Although the literature generally agrees that organic acids followed by simple sugars are often the most common root exudate compounds [27–30], there is considerable variability in these estimates and our artificial solution containing just one representative organic acid and simple sugar is not likely to represent the full suite of potential effects of exudation. Future experiments should measure root exudates *in situ*, characterize the composition, and subsequently use these exudates as substrate in soil incubation experiments.

### Carbon cycle implications

The findings presented in this article have several implications for our understanding of current and future terrestrial C cycling. First, the observation that soil C formation from root exudates is not affected by warming, whereas soil C loss from root exudates is reduced by warming, suggests that the net effect of root exudates on soil C should become more positive in a warmer future. Indeed, we found that the net change in MAOM C over the course of our experiment was less negative in warmed soils. However, our experiment only featured long-term, rather than instantaneous, effects of soil warming (i.e. we extracted soil cores from a long-term warming experiment, but did not keep these cores actively warmed during the exudate addition period). Thus, our experiment tests for the long-term effects of warming on soil microbial community function, rather than the instantaneous effects of temperature on enzymatic velocities. Additionally, our findings likely reflect the stage of the long-term warming experiment. After 20 years of warming, SOM chemistry is altered in the warmed plot relative to the unwarmed plot; enhanced respiration rates under warming over time and more complex C inputs have resulted in more recalcitrant SOM by some metrics [25, 50]. We found evidence of lower soil C quality in the warmed plot than the control plot (Fig. S8), which may have reduced the efficiency of soil microbes carrying out the priming effect. The SOM quality is believed to have exhibited oscillating dynamics over the 20-year course of this experiment, thus, warming may have impacted the priming effect uniquely at different time periods depending on the SOM quality [51].

Our observations of less root exudate-induced soil C loss under long-term soil warming suggest a potential negative climate change feedback: warming induces a shift in the soil microbial community which reduces the root exudate priming effect, resulting in less soil C loss to the atmosphere, thus reducing the greenhouse effect. Many experiments (including this one) have found enhanced soil respiration under active warming [2, 25, 51]—measurements of total CO_2_ efflux from the soil surface already account for any exudate-induced priming. Additionally, soil C stocks were lower in the warmed plot in this experiment than the control plot, so warming still led to an overall C loss [51]. Thus, even though a reduction in the priming effect could attenuate warming-induced respiration increases, it is not likely to fully compensate for them.

Any impact of warming on the root exudate priming effect will also act in tandem with any impact of warming on the root exudation rate. The effects of warming on exudation rates remain complicated, with various experiments reporting positive, negative, or null effects [6–8, 11, 12, 65]. At our field site, exudation rates were reduced in red oak (*Q. rubra*) under warming, but not red maple (*A. rubrum*) [11]. Adding artificial root exudates enhanced soil C loss, so a reduction in exudation rates coupled with a reduction in the exudate-induced priming effect under warming would theoretically mitigate soil C loss. Such a compensatory effect could help explain why soil respiration rates are not always upregulated as much as expected by soil warming [51].

### Conclusion

In this experiment, we show that root exudates induce a priming effect on soil C, likely in part by altering soil microbial community structure and function such that microbes consuming exudate C exhibit different community composition and gene abundances. We show that bacterial community composition and specific microbial gene families are strongly related to the amount of soil C lost to pore-water, an indicator of root exudate-induced priming. Finally, we show that warming reduces the root exudate priming effect, at least in part by altering the soil bacterial community and reducing the abundance of gene families associated with soil C loss, which may be due to adaptation or selection in the bacterial community in response to a lower-quality substrate environment. Our results provide direct evidence that the root exudate priming effect is moderated by the subset of microbes consuming exudate C, and that soil microbes’ capacity to carry out the priming effect may be reduced by future warming climates.

## Supplementary Material

are_2_supplement_wrag002

## Data Availability

The data underlying this manuscript are available at the following DOI: 10.6084/m9.figshare.30297049. The nucleic acid sequences are available under the following GenBank accession number: PRJNA1338260.
