# Correction to: Warming mitigates root exudate-induced priming effects via changes to microbial biomass, community structure, and gene abundance

**DOI:** 10.1093/ismejo/wrag119

**Published:** 2026-06-05

This is a correction to: Nikhil R Chari, Kristen M DeAngelis, Arturo A Aguilar, A Li Han Chan, Grace A Burgin, Serita D Frey, Benton N Taylor, Warming mitigates root exudate-induced priming effects via changes to microbial biomass, community structure, and gene abundance, *The ISME Journal*, Volume 20, Issue 1, January 2026, wrag002, https://doi.org/10.1093/ismejo/wrag002

In the originally published version of the paper there were errors caused in two figures when writing code for figure generation. In Figures. 2b, 2d, and 4a, lettering to indicate significant differences between treatment groups has been corrected to read: “a a b” instead of: “a b b”. The errors do not affect any of the manuscript text. Figure 2 and 4 have been corrected in the paper.

