# Supplementary material for: Warming mitigates root exudate-induced priming effects via changes to microbial biomass, community structure, and gene abundance
Source: ISME J. 2026 Jan 15;20(1):wrag002. doi: 10.1093/ismejo/wrag002 (PMC12904282; doi:10.1093/ismejo/wrag002)
Supplement: are_2_supplement_wrag002 [file are_2_supplement_wrag002.docx]

**Supplementary methods.**

*DNA stable isotope probing.* DNA extracts from samples receiving exudates were ultracentrifuged in a cesium chloride (CsCl) gradient (total 4.5 mL) for 96 h to separate DNA by density. We used a fraction collector to collect the ultracentrifuged samples in ten fractions of 400 uL each, using dyed water to track the sample front as we forced sample out the base of the ultracentrifuge tube (i.e. heavier fractions were collected first). After fraction collection, we measured the density of each fraction using a refractometer. We pooled fractions with <1.4025 nD as a cumulative “light fraction” and fractions with 1.4025-1.4065 nD as a cumulative 13C-enriched “heavy fraction” (there was no or negligible DNA recovered in fractions > 1.4065 nD). We estimated the refraction index cutoff by performing Qubit on all 10 fractions of several samples. Because the heavy fraction samples had much lower amount of DNA than the light fraction, we concentrated these samples using Nanosep filters (Cytiva, Marlborough, MA, USA).

*DNA sequence processing workflows.* For 16S rRNA amplicon sequences, we truncated forward and reverse reads at 150 cycles and filtered using standard parameters [1] with the R package dada2 [2]. We then dereplicated identical sequences, estimated error rates, and inferred real amplicon sequence variants (ASVs) using dada2 [2]. We merged forward and reverse sequence pairs to create an ASV table, removed chimeric sequences using dada2, and assigned taxonomy to ASVs using SILVA v138.1 database [3]. To estimate sequencing depth across samples, we rarefied the ASV table by randomly sub-sampling the counts of each sample to the sample with the smallest number of counts (8111) using the package metaseqR [4]. ASV counts in all samples plateaued before and after rarefaction (Fig. S1), indicating adequate sequencing depth. We combined the ASV table with taxonomy and sample and treatment data to create a phyloseq object for further analyses [5].

We used the bioBakery workflows software to process shotgun metagenomic sequence data and create an enzyme commission (EC) table to determine the abundance of gene families associated with known EC numbers [6]. We trimmed and decontaminated shotgun sequences and paired forward and reverse reads using the kneaddata tool. Then, we used HUMAnN software with the EC-filtered Uniref 90 database to profile gene families with level-4 EC classification and MetaPhlAn software to profile taxonomy [7]. We created a table with all ECs in reads per kilobase (RPK) by sample for downstream analysis. We also specifically annotated CAZy ECs using the online CAZy database [8].

**References.**

1. Edgar RC, Flyvbjerg H. Error filtering, pair assembly and error correction for next-generation sequencing reads. *Bioinformatics* 2015;**31**:3476–3482. https://doi.org/10.1093/bioinformatics/btv401

2. Callahan BJ et al. DADA2: High-resolution sample inference from Illumina amplicon data. *Nature Methods* 2016;**13**:581–583. https://doi.org/10.1038/nmeth.3869

3. Quast C et al. The SILVA ribosomal RNA gene database project: improved data processing and web-based tools. *Nucleic Acids Research* 2013;**41**:D590–D596. https://doi.org/10.1093/nar/gks1219

4. Fanidis D, Moulos P. Integrative, normalization-insusceptible statistical analysis of RNA-Seq data, with improved differential expression and unbiased downstream functional analysis. *Briefings in Bioinformatics* 2021;**22**:bbaa156. https://doi.org/10.1093/bib/bbaa156

5. McMurdie PJ, Holmes S. phyloseq: An R Package for Reproducible Interactive Analysis and Graphics of Microbiome Census Data. *PLOS ONE* 2013;**8**:e61217. https://doi.org/10.1371/journal.pone.0061217

6. McIver LJ et al. bioBakery: a meta’omic analysis environment. *Bioinformatics* 2018;**34**:1235–1237. https://doi.org/10.1093/bioinformatics/btx754

7. Beghini F et al. Integrating taxonomic, functional, and strain-level profiling of diverse microbial communities with bioBakery 3. *eLife* 2021;**10**:e65088. https://doi.org/10.7554/eLife.65088

8. Drula E et al. The carbohydrate-active enzyme database: functions and literature. *Nucleic Acids Research* 2022;**50**:D571–D577. https://doi.org/10.1093/nar/gkab1045

**Supplementary figures and tables.**


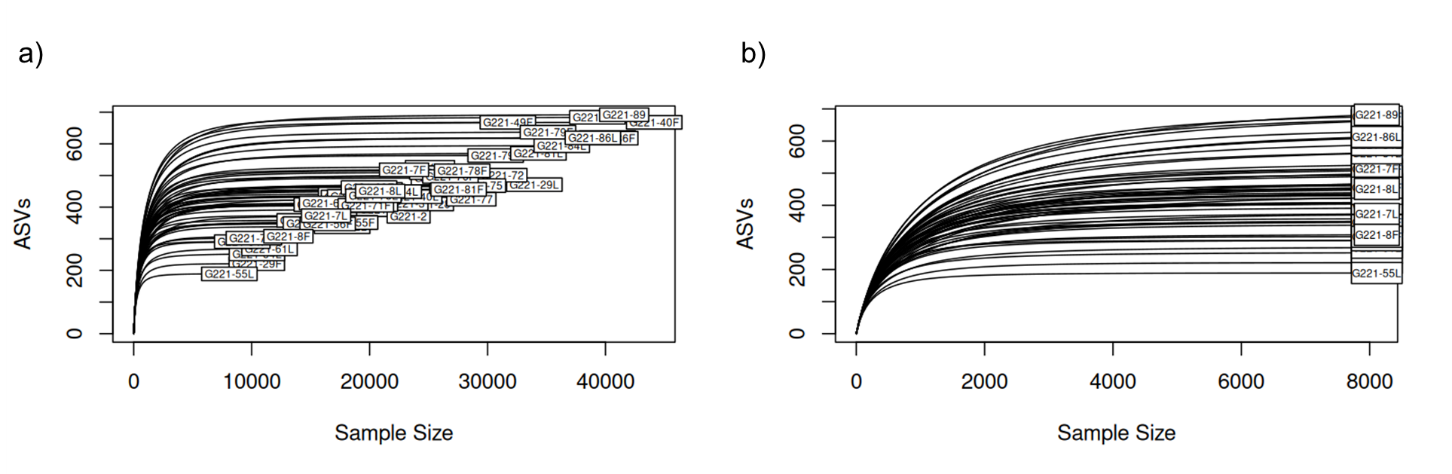


*Fig. S1. ASV diversity curves before (a) and after (b) rarefaction to 8111 counts.*


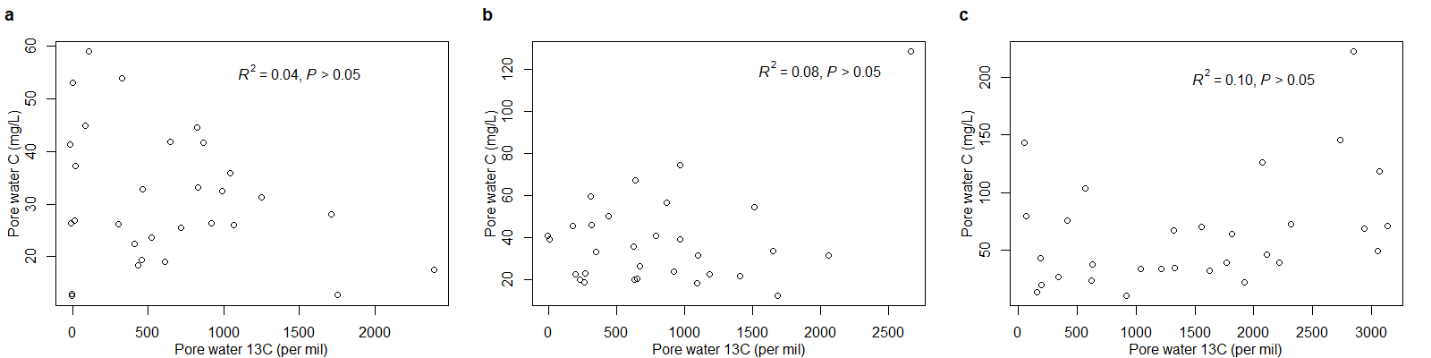


*Fig. S2. No significant relationships were observed between pore water ^13^C and pore water C within any exudation rate treatment, “none” (a), “low rate” (b) and “high rate” (c).*


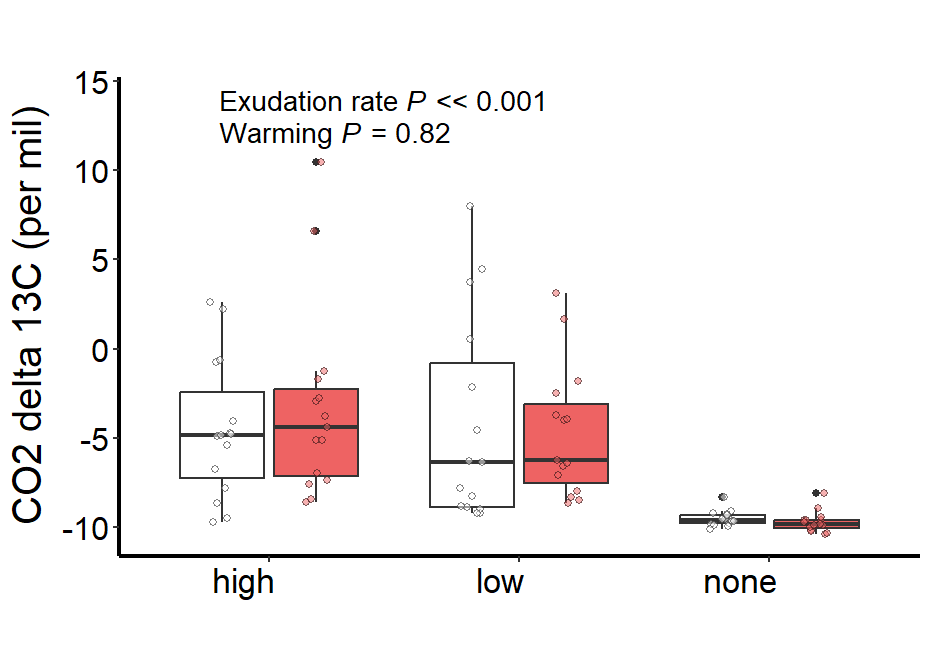


*Fig. S3. Effect of exudation rate and warming treatments on respiration of ^13^CO_2_. The horizontal line is the median and the rectangles are the interquartile range, and the bars are the minimum and maximum, excluding outliers, which are marked by black points.*


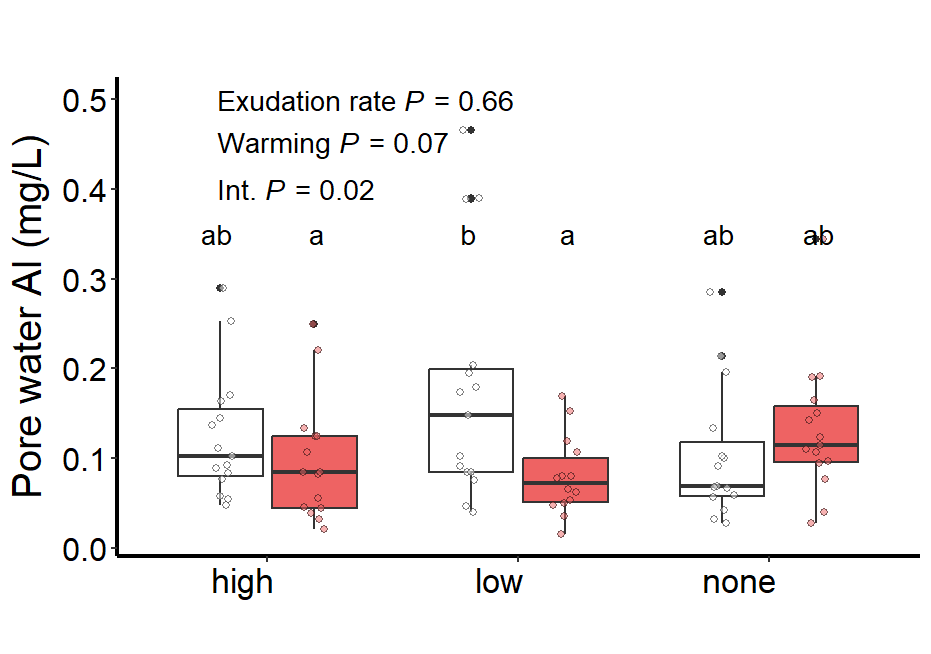


*Fig. S4. Effect of exudation rate and warming treatments on pore water aluminum concentration. The horizontal line is the median and the rectangles are the interquartile range, and the bars are the minimum and maximum, excluding outliers, which are marked by black points.*

*
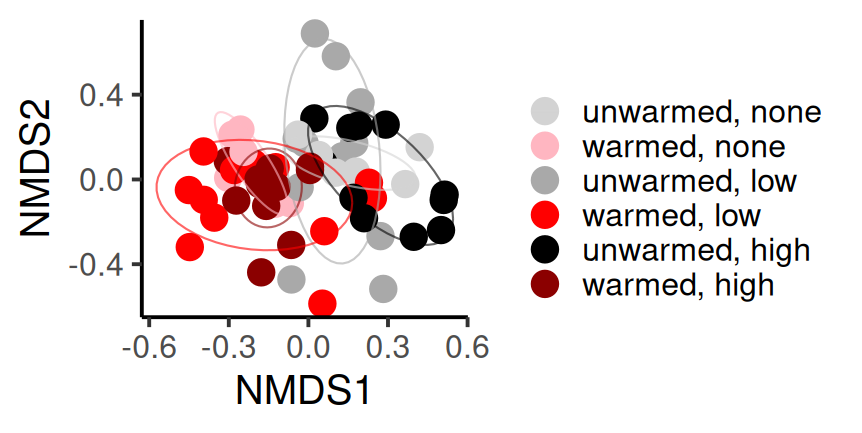
*

*Fig. S5. NMDS plot showing interaction between warming and exudation rate treatments on microbial ASV composition. The interaction effect is from “warmed, low” and “warmed, high” being statistically different groupings but “unwarmed, low” and “unwarmed, high” not.*


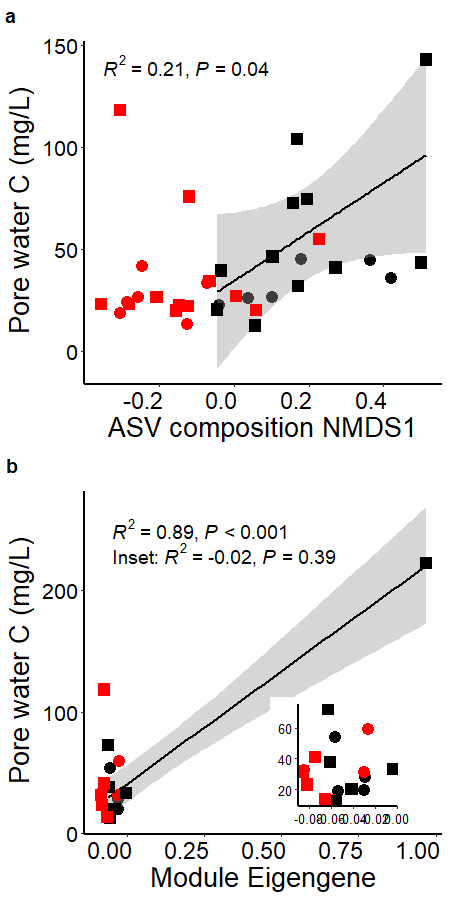


*Fig. S6. Relationships shown in main text Fig. 5 but with light fraction samples (not enriched in exudate ^13^C) instead of heavy fraction samples (enriched in exudate ^13^C). Both relationships are weaker than those shown in the main text.*


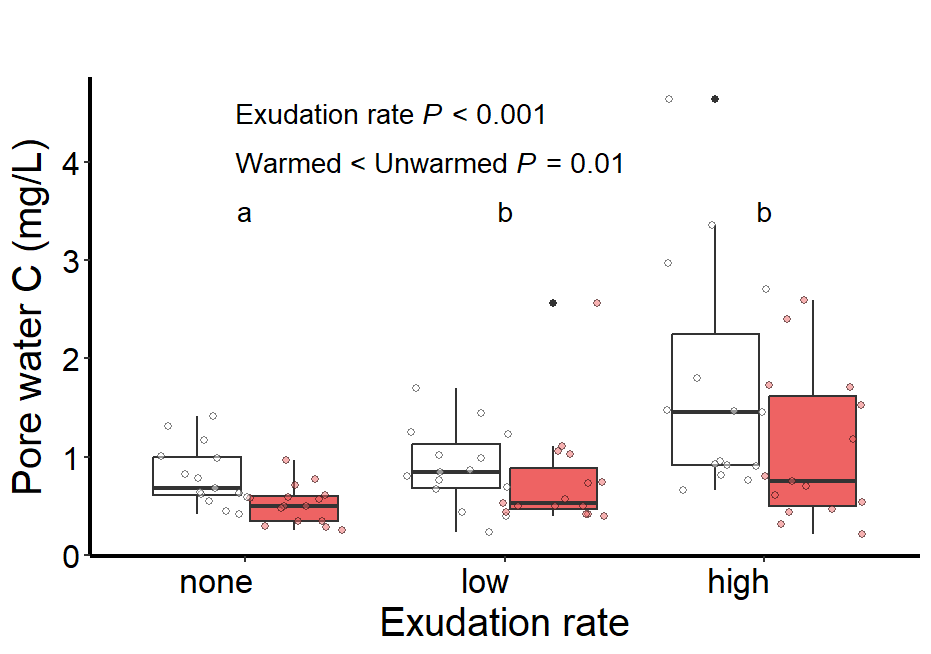


*Fig. S7. Total pore water C in response to exudation rate and warming treatments. Total pore water C is pore water C in mg L^-1^ (shown in Fig. 1b) scaled by the total amount of water in the soil core.*


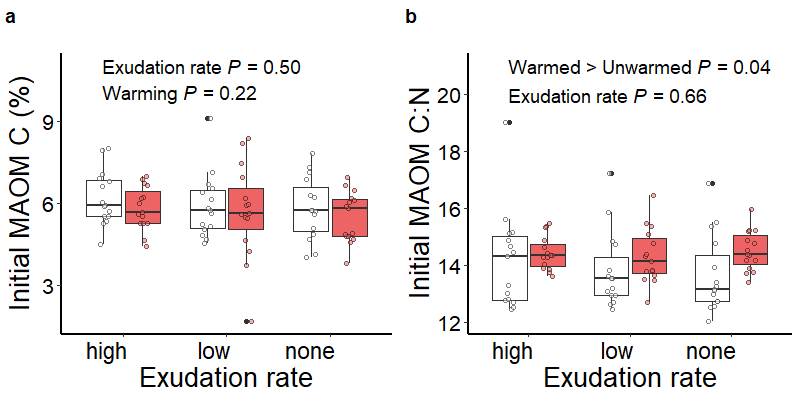


*Fig. S8. Initial MAOM C (a) and MAOM C:N ratio (b) from replicate cores.*

**Table S1.** Phylum response to warming and in ^13^C-enriched “heavy” isotope fraction. Only phyla that respond significantly are listed, in order of abundance

| **Warmed:Unwarmed** | **Heavy:Light** | **Heavy:None** | **Light:None** |
| --- | --- | --- | --- |
| Acidobacteriota (-) | Proteobacteria (+) | Proteobacteria (+) | Crenarchaeota (-) |
| Verrucomicrobiota (+) | Actinobacteriota (+) | Verrucomicrobiota (-) | Dadabacteria (-) |
| Actinobacteriota (+) | Chloroflexi (+) | Actinobacteriota (+) |  |
| Chloroflexi (+) | Bacteroidota (-) | Chloroflexi (+) |  |
| Crenarchaeota (+) | Myxococcota (+) | Gemmatimonadota (+) |  |
| Eremiobacterota (+) | Methylomirabilota (+) | Bdellovibrionota (-) |  |
| Candidate phylum WPS-2 (+) | Gemmatimonadota (+) | Candidate phylum FCPU426 (-) |  |
| Candidate phylum NB1-j (+) | Desulfobacterota (+) |  |  |
| Latescibacterota (-) | Dependentiae (-) |  |  |
| Candidate phylum GAL15 (+) | Armatimonadota (+) |  |  |
| Candidate phylum SAR324 (-) | Candidate phylum MBNT15 (+) |  |  |
|  | Thermoplasmatota (+) |  |  |

**Table S2.** List of EC gene families from WGCNA module that was correlated with pore water C

| **EC number** | **Enzyme name** | **KEGG pathways** |
| --- | --- | --- |
| 1.1.1.208 | (+)-neomenthol dehydrogenase | Biosynthesis of secondary metabolites  Monoterpenoid biosynthesis |
| 1.1.1.219 | dihydroflavonol 4-reductase | Metabolic pathways  Biosynthesis of secondary metabolites  Flavonoid biosynthesis |
| 1.1.1.264 | L-idonate 5-dehydrogenase |  |
| 1.1.1.289 | sorbose reductase | Metabolic pathways  Fructose and mannose metabolism |
| 1.1.1.302 | 2,5-diamino-6-(ribosylamino)-4(3H)-pyrimidinone 5'-phosphate reductase | Metabolic pathways  Riboflavin metabolism |
| 1.1.1.325 | sepiapterin reductase (L-threo-7,8-dihydrobiopterin-forming) | Metabolic pathways  Folate biosynthesis |
| 1.1.1.88 | hydroxymethylglutaryl-CoA reductase | Biosynthesis of secondary metabolites  Terpenoid backbone biosynthesis |
| 1.1.5.12 | D-lactate dehydrogenase (quinone) | Metabolic pathways  Pyruvate metabolism |
| 1.14.13.172 | salicylate 5-hydroxylase | Metabolic pathways  Microbial metabolism in diverse environments  Naphthalene degradation |
| 1.2.1.5 | aldehyde dehydrogenase [NAD(P)+] | Metabolic pathways  Biosynthesis of secondary metabolites  Microbial metabolism in diverse environments  Drug metabolism - cytochrome P450  Phenylalanine metabolism  beta-Alanine metabolism  Glycolysis/Gluconeogenesis  Histidine metabolism  Metabolism of xenobiotics by cytochrome P450  Tyrosine metabolism |
| 1.2.1.65 | salicylaldehyde dehydrogenase | Metabolic pathways  Microbial metabolism in diverse environments  Naphthalene degradation |
| 1.2.7.3 | 2-oxoglutarate synthase | Metabolic pathways  Biosynthesis of secondary metabolites  Microbial metabolism in diverse environments  Citrate cycle (TCA cycle)  Other carbon fixation pathways |
| 1.2.98.1 | formaldehyde dismutase | Metabolic pathways  Microbial metabolism in diverse environments  Methane metabolism  Chloroalkane and chloroalkene degradation |
| 1.21.4.1 | D-proline reductase | Metabolic pathways  Arginine and proline metabolism  D-amino acid metabolism |
| 1.3.1.3 | Delta4-3-oxosteroid 5beta-reductase | Metabolic pathways  Steroid hormone biosynthesis  Primary bile acid biosynthesis |
| 1.3.1.56 | cis-2,3-dihydrobiphenyl-2,3-diol dehydrogenase | Metabolic pathways  Microbial metabolism in diverse environments  Dioxin degradation |
| 1.3.1.58 | 2,3-dihydroxy-2,3-dihydro-p-cumate dehydrogenase | Metabolic pathways  Microbial metabolism in diverse environments  Xylene degradation |
| 1.3.98.1 | dihydroorotate dehydrogenase (fumarate) | Metabolic pathways  Pyrimidine metabolism |
| 2.1.1.176 | 16S rRNA (cytosine967-C5)-methyltransferase |  |
| 2.1.1.181 | 23S rRNA (adenine1618-N6)-methyltransferase |  |
| 2.1.1.288 | aklanonic acid methyltransferase | Metabolic pathways  Biosynthesis of secondary metabolites  Biosynthesis of type II polyketide products |
| 2.1.1.37 | DNA (cytosine-5-)-methyltransferase | Metabolic pathways  Cysteine and methionine metabolism |
| 2.1.3.10 | malonyl-S-ACP:biotin-protein carboxyltransferase |  |
| 2.3.1.18 | galactoside O-acetyltransferase |  |
| 2.3.1.28 | chloramphenicol O-acetyltransferase |  |
| 2.3.1.30 | serine O-acetyltransferase | Metabolic pathways  Biosynthesis of secondary metabolites  Microbial metabolism in diverse environments  Cysteine and methionine metabolism  Sulfur metabolism  Biosynthesis of various antibiotics |
| 2.4.1.173 | sterol 3beta-glucosyltransferase (**CAZyme**) |  |
| 2.5.1.47 | cysteine synthase | Metabolic pathways  Biosynthesis of secondary metabolites  Microbial metabolism in diverse environments  Cysteine and methionine metabolism  Sulfur metabolism  Sulfur cycle |
| 2.5.1.78 | 6,7-dimethyl-8-ribityllumazine synthase | Metabolic pathways  Biosynthesis of secondary metabolites  Riboflavin metabolism |
| 2.5.1.9 | riboflavin synthase | Metabolic pathways  Biosynthesis of secondary metabolites  Riboflavin metabolism |
| 2.6.99.3 | O-ureido-L-serine synthase | Metabolic pathways  Biosynthesis of secondary metabolites  Biosynthesis of various antibiotics |
| 2.7.1.144 | tagatose-6-phosphate kinase | Metabolic pathways  Galactose metabolism |
| 2.7.1.170 | anhydro-N-acetylmuramic acid kinase | Metabolic pathways  Amino sugar and nucleotide sugar metabolism |
| 2.7.11.1 | non-specific serine/threonine protein kinase |  |
| 2.7.4.1 | ATP-polyphosphate phosphotransferase | Oxidative phosphorylation |
| 2.7.7.59 | [protein-PII] uridylyltransferase |  |
| 2.7.8.28 | 2-phospho-L-lactate transferase | Metabolic pathways  Microbial metabolism in diverse environments  Methane metabolism |
| 2.7.8.36 | undecaprenyl phosphate N,N'-diacetylbacillosamine 1-phosphate transferase |  |
| 2.7.8.8 | CDP-diacylglycerol---serine O-phosphatidyltransferase | Metabolic pathways  Biosynthesis of secondary metabolites  Glycerophospholipid metabolism  Glycine, serine, and threonine metabolism |
| 3.2.2.28 | double-stranded uracil-DNA glycosylase |  |
| 3.4.11.25 | beta-peptidyl aminopeptidase |  |
| 3.4.13.22 | D-Ala-D-Ala dipeptidase |  |
| 3.4.17.13 | muramoyltetrapeptide carboxypeptidase |  |
| 3.4.21.102 | C-terminal processing peptidase |  |
| 3.5.1.n3 | 4-deoxy-4-formamido-L-arabinose-phosphoundecaprenol deformylase |  |
| 3.6.1.1 | inorganic diphosphatase | Oxidative phosphorylation |
| 3.6.3.19 | ABC-type maltose transporter |  |
| 3.6.3.38 | ABC-type capsular-polysaccharide transporter |  |
| 3.6.3.6 | P-type H+-exporting transporter |  |
| 3.8.1.10 | 2-haloacid dehalogenase (configuration-inverting) |  |
| 4.2.1.109 | methylthioribulose 1-phosphate dehydratase | Metabolic pathways  Cysteine and methionine metabolism |
| 4.2.1.129 | squalene---hopanol cyclase | Biosynthesis of secondary metabolites  Sesquiterpenoid and triterpenoid biosynthesis |
| 4.2.1.171 | cis-L-3-hydroxyproline dehydratase | Arginine and proline metabolism |
| 4.2.1.47 | GDP-mannose 4,6-dehydratase | Metabolic pathways  Fructose and mannose metabolism  Biosynthesis of various nucleotide sugars |
| 4.2.3.12 | 6-pyruvoyltetrahydropterin synthase | Metabolic pathways  Folate biosynthesis |
| 4.99.1.3 | sirohydrochlorin cobaltochelatase | Metabolic pathways  Porphyrin metabolism |
| 5.1.1.20 | L-Ala-D/L-Glu epimerase |  |
| 5.1.3.30 | D-psicose 3-epimerase |  |
| 5.3.1.30 | 5-deoxy-glucuronate isomerase | Metabolic pathways  Microbial metabolism in diverse environments  Inositol phosphate metabolism |
| 5.3.99.11 | 2-keto-myo-inositol isomerase | Metabolic pathways  Microbial metabolism in diverse environments  Inositol phosphate metabolism |
| 5.4.99.12 | tRNA pseudouridine38-40 synthase |  |
| 5.5.1.4 | inositol-3-phosphate synthase | Metabolic pathways  Biosynthesis of secondary metabolites  Inositol phosphate metabolism  Streptomycin biosynthesis |
| 6.2.1.12 | 4-coumarate---CoA ligase | Metabolic pathways  Biosynthesis of secondary metabolites  Phenylpropanoid biosynthesis  Ubiquinone and other terpenoid-quinone biosynthesis |
| 6.3.3.3 | dethiobiotin synthase | Metabolic pathways  Biotin metabolism |
| 6.3.3.5 | O-ureido-D-serine cyclo-ligase | Metabolic pathways  Biosynthesis of secondary metabolites  Biosynthesis of various antibiotics |
| 6.3.4.2 | CTP synthase (glutamine hydrolysing) | Metabolic pathways  Pyrimidine metabolism |
| 7.3.2.3 | ABC-type sulfate transporter | Sulfur metabolism |
